# *Adamts18* deficiency promotes colon carcinogenesis by enhancing β-catenin and p38MAPK/ERK1/2 signaling in the mouse model of AOM/DSS-induced colitis-associated colorectal cancer

**DOI:** 10.18632/oncotarget.14866

**Published:** 2017-01-27

**Authors:** Tiantian Lu, Suying Dang, Rui Zhu, Ying Wang, Zongying Nie, Tao Hong, Wei Zhang

**Affiliations:** ^1^ Key Laboratory of Brain Functional Genomics, Ministry of Education, Shanghai Key Laboratory of Brain Functional Genomics, School of Life Science, East China Normal University, Shanghai, China; ^2^ Department of Biochemistry and Molecular Cell Biology, Shanghai Jiao Tong University School of Medicine, Shanghai, China; ^3^ Shanghai Research Center for Model Organisms, Shanghai, China

**Keywords:** ADAMTS18, colitis-associated colorectal cancer, inflammation, tumorigenesis

## Abstract

ADAMTS18 is a novel tumor suppressor and is critical to the pathology of human colorectal cancer. However, the underlying mechanism is not clear. Here we generated an *Adamts18*-deficient mouse strain as an *in vivo* model to investigate the role of ADAMTS18 in the pathogenesis of colorectal cancer. In AOM/DSS–induced colitis-associated colorectal cancer, the deficiency of *Adamts18* in mice resulted in enhanced tumorigenesis and colon inflammation that could be attributed in part to enhanced nuclear translocation of β-catenin and elevated expression of its downstream target genes, cyclin D1 and c-myc. Moreover, increased p38MAPK and ERK1/2 activities were detected in colon cancer cells from *Adamts18*-deficient mice. Further studies revealed that ADAMTS18 deficiency reduced intestinal E-cadherin levels in mice, which ultimately led to intestinal barrier dysfunction. These data indicate that Adamts18 deficiency enhances tumorigenesis and intestinal inflammation through elevated Wnt/β-catenin and p38MAPK/ERK1/2 signaling and promotes colon cancer in this mouse model.

## INTRODUCTION

A disintegrin and metalloproteinase with thrombospondin motifs (ADAMTSs) are a family of 19 secreted zinc-metalloendopeptidases, which have been implicated in development, tissue remodeling, inflammation, cancer and vascular biology. ADAMTS proteases have multi-domain structural components in common, including an N-terminal signal peptide, followed by a prodomain, a metalloproteinase catalytic domain with a zinc binding motif, a disintegrin-like domain, a central thrombospondin type-1-like repeat (TSR), a cysteine-rich domain (high sequence homology), a spacer region, and a variable number of C-terminal TSR repeats. Some of them have further C-terminal domains. These enzymes are located at the pericellular space through interactions of their thrombospondin-1 motifs and spacer regions with extracellular matrix (ECM) components [1, 2].

ADAMTS18 is an orphan ADAMTS, whose physiological substrates have not been identified. This metalloproteinase is widely detected in adult tissues including brain, prostate, submaxillary gland, endothelium, retina, heart, lung, skeletal muscle, spleen, pancreas, esophagus, stomach, colon, larynx, breast, cervix, placenta, ovary, bone marrow, and lymph nodes [2]. The top five tissues where ADAMTS18 is most highly expressed are the cerebellar vermis, cerebellum, cerebellar hemisphere, transverse colon, and the corpus callosum (NextBio Body Atlas; nextbio.com). Mutations of *ADAMTS18* have been linked to eye diseases [3–5], bone mineral density (BMD) formation [6], white matter integrity of the brain [7], and various malignancies [8–12].

The potential role of ADAMTS18 in tumorgenesis is first suggested by the genetic linkage analysis. It has been shown that the loss of heterozygosity assay of 16q23 region is strongly associated with a variety of cancers [8–10]. Since ADAMTS18 is one of these genes located around 16q23 region, it has been studied as a potential tumor suppressor gene. Mutational analysis of human breast and colorectal carcinomas highlighted the occurrence of some mutations in *ADAMTS18* [11]. Furthermore, this enzyme was found to be frequently methylated inactivation in multiple tumor cell lines or carcinomas [12]. We hypothesize that ADAMTS18 plays protective roles in colon carcinogenesis *in vivo*. To investigate how ADAMTS18 exerts its effect on colorectal cancer as well as the involved molecular events, we generate *Adamts18* gene knockout (KO) mice and demonstrate that ADAMTS18 deficiency creates a tumor-promoting microenvironment for colon tumor formation in mice.

## RESULTS

### Generation of *Adamts18* deficient mice

**Figure 1:**
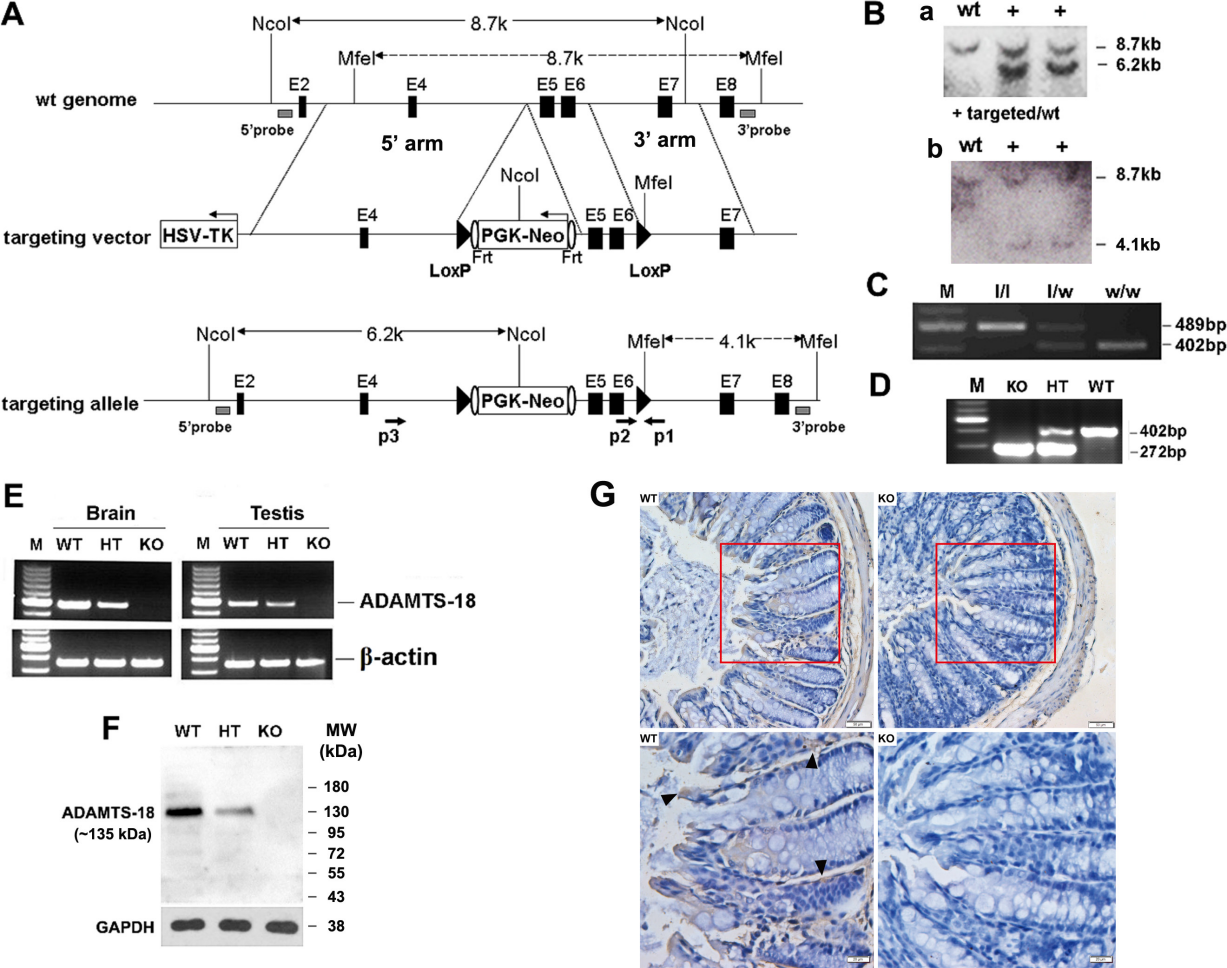
Targeted disruption of mouse *Adamts18* gene (**A**) Schematic illustration of the gene-targeting strategy. In the targeting vector, a Frt (shown as vertical oval) flanked PGK/Neo cassette which was put into the intron upstream of exon5 and exons 5–6 were flanked by two loxP sites (black triangles), and a HSV-TK cassette was introduced in the flanking region of the 5′ arm. Black horizontal bar, linear genomic sequence of the *Adamts18* gene; Filled box, exon; HSV-TK, herpes simplex virus thymidine kinase; Frt, flippase recognition target site. (**B**) Southern blot analysis of genomic DNA from ES cell clones. (a) Genomic DNA from ES cell clones were digested with NcoI and hybridized with a 5′probe located in the flanking region of 5′homologous arm (shown in panel A as gray box). (b) Another Southern blotting with MfeI-digested genomic DNA. (**C**) Example of PCR genotyping of *Adamts18* floxed (l/l), *Adamts18* floxed heterozygous (l/w), and WT (w/w) mice with p1 and p2 (shown as opposite arrows in panel A) located in the flanking regions of the introduced loxP downstream of the exon 6. (**D**) Example of PCR genotyping of *Adamts18* knockout (KO), heterozygous (HT), and wild type (WT) mice. (**E**) RT-PCR analysis for *Adamts18* mRNA in brain and testis of WT, HT and KO mice. β-actin was used as internal reference. M, DNA molecular marker. (**F**) Western blot analysis of Adamts18 protein expression in brain of WT, HT and KO mice. GAPDH was used as the loading control. (**G**) Immunohistochemical staining. A strong *Adamts18* expression was noted in the mucosa (black arrowhead) of WT mouse colon when compared with *Adamts18* KO littermates. Scale bar = 50 μm (upper panel) and 20 μm (lower panel), respectively.

The targeting vector was designed to delete exons 5–6 of *Adamts18* gene which correspond to the N-terminal part of the catalytic domain of the Zinc-dependent metalloprotease and to create a frame shift mutation after the deletion (Figure 1A). To circumvent potential embryonic lethality, we initially generated a mouse strain with LoxP flanked (exons 5–6) *Adamts18* gene allele. Homologous recombination was identified by Southern blotting in two embryonic stem (ES) clones. The expected 8.7 kb (shown in panel A) band of wild type allele and 6.2 kb (shown in panel A) band from targeted allele were detected in two ES cell clones (Figure 1B, a). Another Southern blotting with MfeI-digested genomic DNA was hybridized with a 3′probe located in the flanking region of 3′arm (shown in panel A as gray box), and the 8.7 kb (shown in panel A) WT fragment and the 4.1kb (shown in panel A) targeted fragment were detected in the same two ES cell clones (Figure 1B, b). Germ line transmission of the LoxP flanked (exons 5–6) *Adamts18* gene allele was verified by Southern blotting (data not shown) and polymerase chain reaction (PCR). A 402-bp fragment was amplified from wild type (w/w) mice; a 489-bp fragment was amplified from *Adamts18* floxed (l/l) mice; and both the 402-bp and 489-bp fragments were amplified from *Adamts18* floxed heterozygous (l/w) mice (Figure 1C). Germ line deletion of exons 5–6 of *Adamts18* gene was generated by crossing the *Adamts18* floxed mice with Sox2-Cre mice and was identified by genome DNA PCR. A 402-bp fragment was amplified with p1 and p2 from wild type (WT) mice; a 272-bp fragment was amplified with p3 (located in 5′flanking region of the introduced loxP between exon4 and exon5, and shown as rightward arrow in panel A) and p1 from homozygous *Adamts18* KO mice; and both the 402-bp and 272-bp fragments were amplified from *Adamts18* heterozygous (HT) mice (Figure 1D). The sequencing result of the PCR product from *Adamts18* KO mice verified the deletion of DNA between the two Loxp sites (data not shown). The absence of *Adamts18* transcript from exons 4–6 in *Adamts18* KO mice was determined by semi-quantitative reverse transcriptase RT-PCR. The 472-bp transcript amplified with the primer pair (E_4_F and E_6_R) specific to mouse *Adamts18* exon4 and exon6 was absent in *Adamts18* KO mouse RNA (Figure 1E). The absence of ADAMTS18 protein (~135 KDa) was confirmed via analyzing the brain proteins by Western blot analysis (Figure 1F). In addition, ADAMTS18 expression was observed in the mucosa of mouse colon by immunohistochemical staining (Figure 1G). *Adam*ts18 KO mice with a mixed 129Sv/C57BL/6 background were viable and exhibit normal survival. However, small sizes and abnormal eye development as well as expanded alveoli had been observed in a substantial fraction of *Adamts18* KO mice (unpublished data), which is similar to the recent reported *Adamts18* null mouse with C57Bl6/Ola background that have distinct developmental defects including disorders of lens, lung, and female reproductive tract development [13].

### *Adam*ts18 deficiency enhances tumorigenesis and intestinal inflammation in the murine AOM/DSS–induced colitis-associated colon cancer (CAC) model

**Figure 2:**
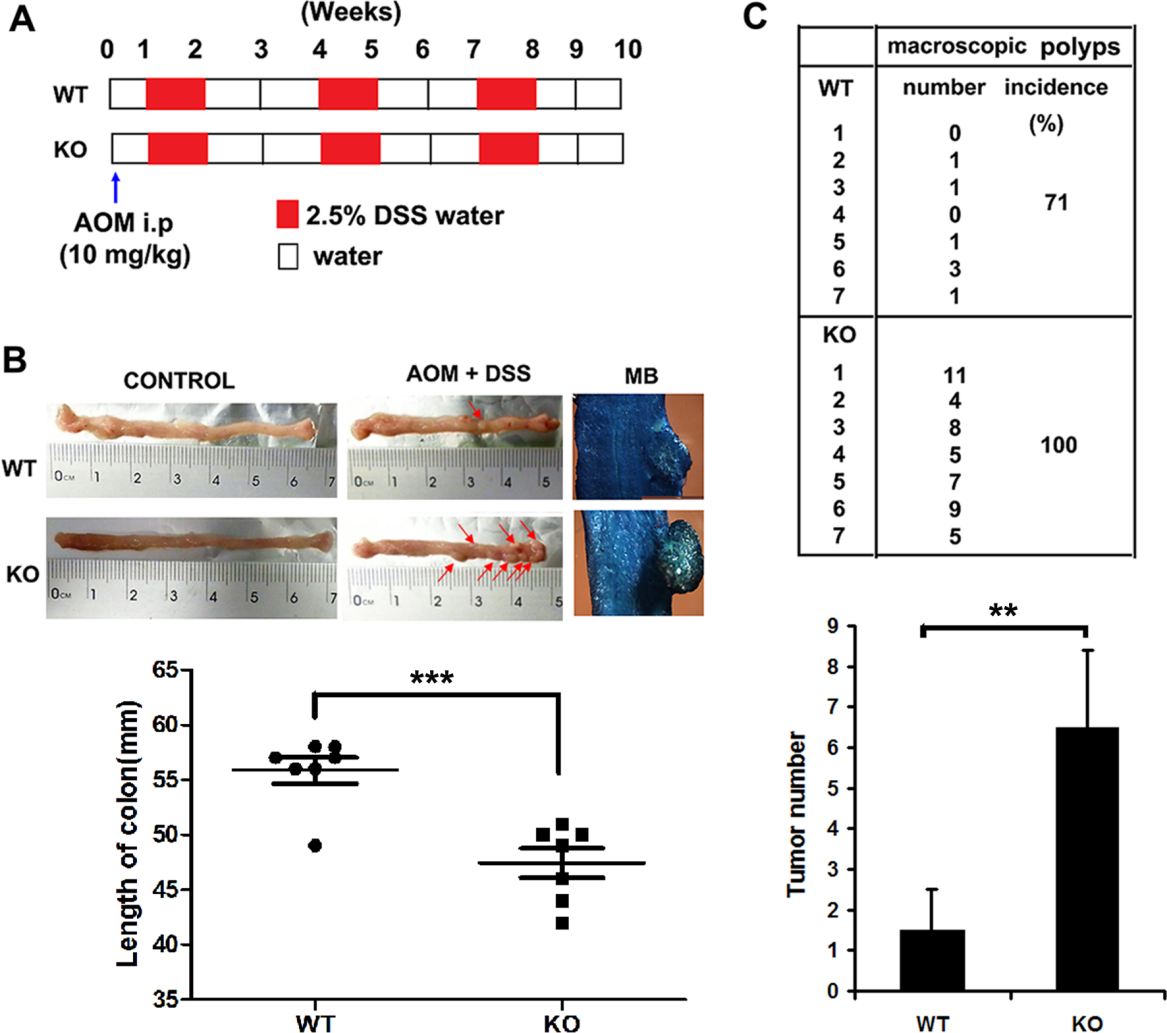
AOM/DSS-induced colitis-associated colon cancer in mice (**A**) Study design. The age- and sex-matched wild-type (WT) mice (*n* = 7) and *Adamts18* knock-out (KO) littermates (*n* = 7) were treated with AOM (10 mg/kg) and then received three rounds of 2.5% DSS exposure. At week 10, all mice were sacrificed for further pathological analysis. (**B**) The formation of colorectal tumors in the distal part of the colon after AOM/DSS induction (upper panel). Control, representative photos of normal colon from both WT and *Adamts18* KO mice. Red arrow, macroscopic polyps in colons. MB, representative images of intestinal wall polyps stained by methylene blue (scale bar = 1 mm). Lower panel, quantification of colon length (***P < 0.001). (**C**) Number and incidence of macroscopic polyps in WT and *Adamts18* KO mice (upper panel); Lower panel, quantification of tumor numbers (**P < 0.01). Columns mean; bars, SD. These data are representative of three separate experiments.

*Adamts18* KO mice and the age- and sex-matched wild-type littermates were injected with the procarcinogen azoxymethane (AOM) and then received three rounds of dextran sodium sulfate (DSS) exposure to elicit colitis (Figure 2A). Mice treated with AOM plus DSS developed tumors mainly located in the distal part of the colon (Figure 2B, upper panel). Significantly reduced colon lengths, a symptom of inflammation, were also observed in *Adamts18* KO mice when compared with WT littermates (WT *vs*. KO, 55.8 ± 3.1 *vs*. 47.4 ± 3.4 mm, *P* = 0.0004) (Figure 2B, lower panel). *Adamts18* KO mice demonstrated higher tumor incidence rates and more tumor numbers than WT littermates (macroscopic polyps, WT *vs*. KO, 71% *vs*. 100%; tumor numbers, WT *vs*. KO, 1.5 ± 1 *vs*. 6.5 ± 1.9; *n* = 7/group, *P* = 0.0026) (Figure 2C).

**Figure 3:**
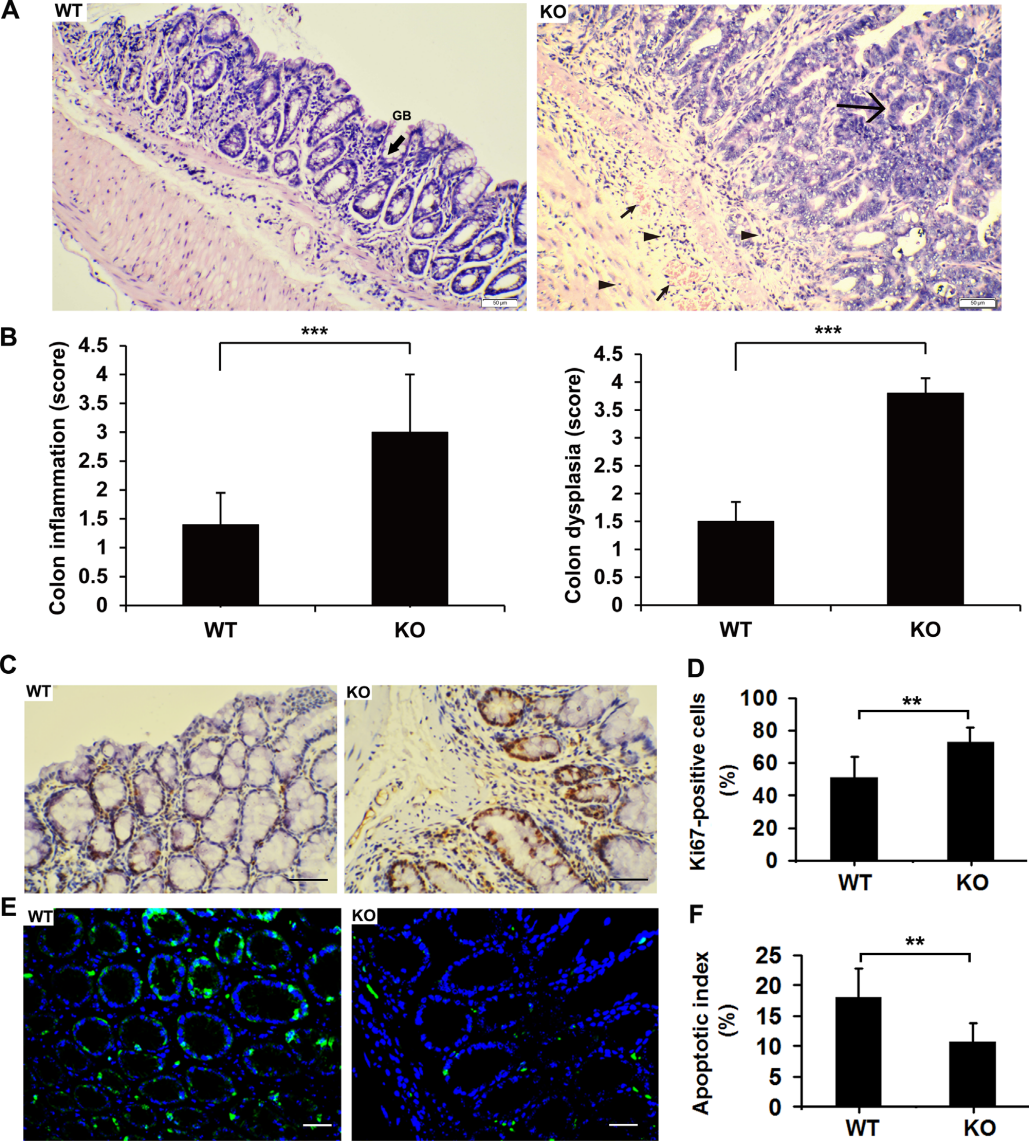
Enhanced tumorigenesis and intestinal inflammation in Adamts18 KO mice (**A**) Hematoxylin and eosin (HE) staining of representative colonic sections. Left panel, colonic sections of WT mice after AOM/DSS treatment. GB, refers to goblet cells (black arrow sign). Right panel, marked histopathological changes were seen in colonic sections of *Adamts18* KO mice characterized by severe dysplasia in the mucosa with loss of the gland structures (large arrow sign), inflammatory cell infiltrate around the crypt base and diffuse into the submucosa (small triangle arrows) as well as submucosa congestion (small arrow sign) when compared to WT littermates. (**B**) Histological inflammation and dysplastic scores determined by specialist in a double-blind fashion as described in methods. (C-D) Ki-67 immunohistochemistry staining (**C**) and percentage of Ki-67-positive cells (**D**). (**E**–**F**) Tumor cell apoptosis was assessed using TUNEL apoptosis detection kit (green). DAPI (blue) indicates nucleus. TUNEL, TdT-mediated dUTP nick end labeling; DAPI, 4, 6-diamidino-2-phenylindole. Scale bar = 50 μm. Columns mean; bars, SD (**P < 0.01; ***P < 0.001). These experiments are repeated independently at three times.

Histological examination revealed that the numbers of goblet cells (GB), which secrete mucus to protect the mucous membranes, were significantly decreased in colonic sections of *Adamts18* KO mice relative to WT littermates (Figure 3A). Furthermore, marked histopathological changes were seen in colonic sections of *Adamts18* KO mice characterized by severe dysplasia in the mucosa with loss of the gland structures, inflammatory cell infiltrate around the crypt base and diffuse into the submucosa as well as submucosa congestion when compared to WT littermates (Figure 3A, right panel). Pathological score based on the degree of inflammation and dysplasia in *Adamts18* KO mice were higher than WT littermates (Figure 3B). Consistent with the changes in colon tumor burden, the number of cells expressing Ki-67 was significantly more in colon tumors from *Adamts18* KO mice in comparison to WT tumor cells [Ki-67 positive cells (%), WT *vs*. KO, 50.8 ± 12.9 *vs*. 72.7 ± 9.2; *P* = 0.003] (Figure 3C and 3D). This suggests *Adamts18* deficiency promotes proliferation of tumor cells. In addition, the results of *in situ* TUNEL assay showed *Adamts18* KO tumor cells underwent less apoptosis than WT tumor cells [apoptotic index (%), WT *vs*. KO, 18.1 ± 4.7 *vs*. 10.7 ± 3.1; *P* = 0.0044] (Figure 3E and 3F).

Since some ADAMTSs (e.g. ADAMTS-1 and -8) have anti-angiogenic activities that contribute to the inhibition of cancer development [14, 15], we thus examined the relevance of *Adamts18* with tumor angiogenesis. We didn't find significant difference in microvessel densities of colon tumors (Supplementary Figure 1) and the expression of *Adamts1, 8, 12* which were reported involved in angiogenesis (Supplementary Table 1) between *Adamts18* KO mice and WT littermates. Thus, the enhanced tumorgenesis in *Adamts18* KO mice was not related to angiogenic effect.

### Enhanced nuclear translocation of β-catenin in colon cancer cells from *Adamts18*-deficient mice

**Figure 4:**
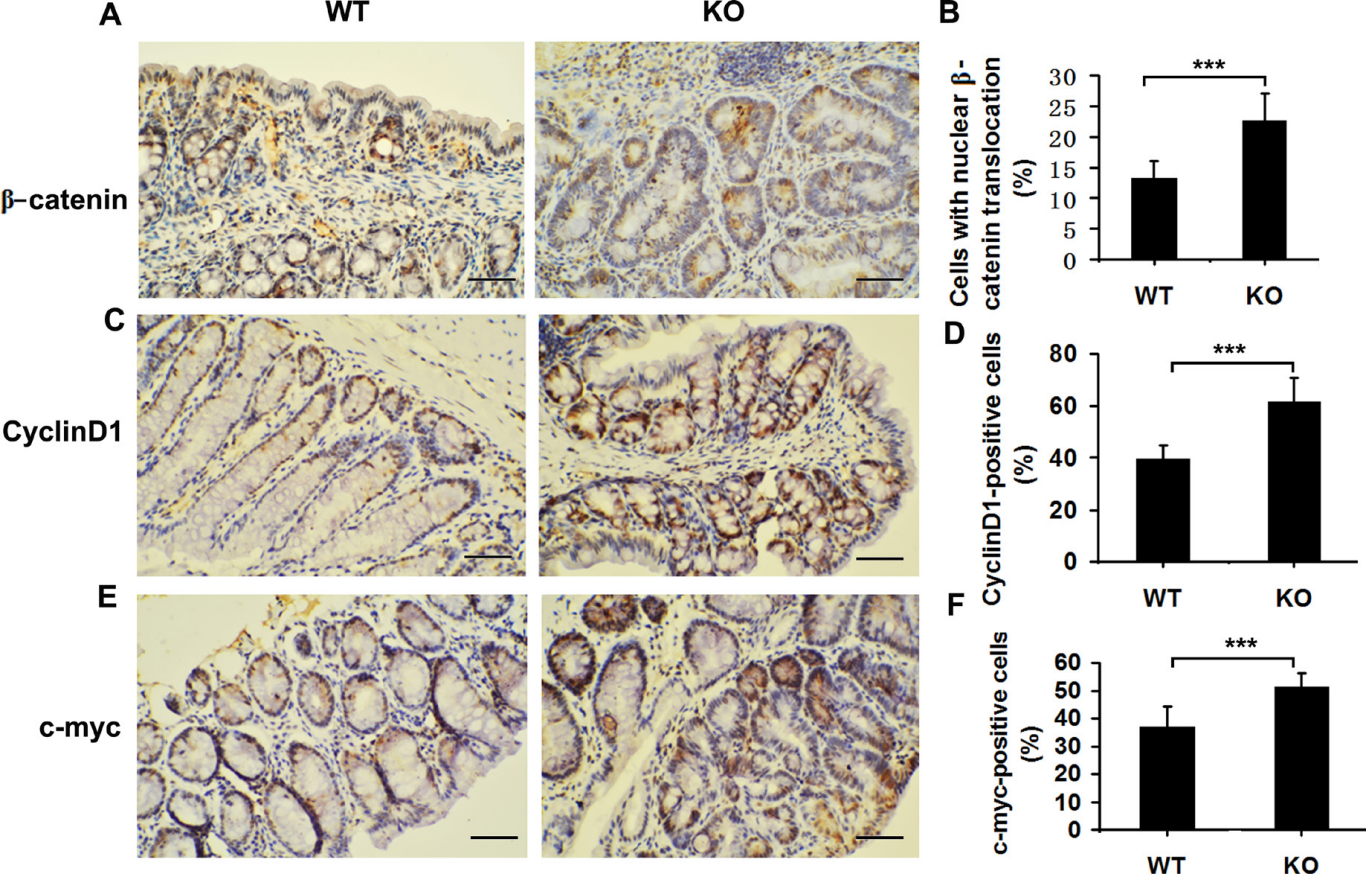
Elevated Wnt/β-catenin signaling pathway in Adamts18 deficient mice (**A**, **C**, **E**) β-catenin (A), cyclinD1 (C), and c-myc (E) immunohistochemistry staining. (**B**, **D**, **F**) Percentage of β-catenin-positive (B), cyclinD1-positive (D), and c-myc-positive (F) tumor cells. Brown, positive staining. Columns, mean; bars, SD (**P < 0.01; ***P < 0.001). Scale bar = 50 μm. These experiments are repeated independently at three times.

In AOM/DSS–induced CAC, enhanced tumorigenesis is closely associated with aberrant β-catenin signaling pathway [16]. We then examined the association between *Adamts18* and β-catenin signaling pathway. Immunohistochemistry staining showed that the number of cells with nuclear β-catenin translocation was significantly increased in *Adamts18* KO tumor cells compared to WT tumor cells [cells with nuclear β-catenin translocation (%), WT *vs*. KO, 13.1 ± 2.9 *vs*. 22.7 ± 4.3; *P* = 0.0004] (Figure 4A and 4B). Translocated β-catenin from the cytoplasm to the nucleus may serve as a transcriptional factor to stimulate the expression of its downstream target genes (cyclinD1 and c-myc) and promote colon tumor formation [16]. In line with this views, we found that the numbers of cyclinD1 or c-myc- positive cells were also significantly increased in colon tumors from *Adamts18* KO mice in comparison to WT tumor cells [cyclinD1 positive cells (%), WT *vs*. KO, 39.4 ± 5.4 *vs*. 61.3 ± 9.5; *P* = 0.0002; c-myc positive cells (%), WT *vs*. KO, 36.8 ± 7.4 *vs*. 51.4 ± 4.5; *P* = 0.0008] (Figure 4C–4F). Likewise, the results of qRT-PCR showed the expression of its downstream target genes cyclinD1 and c-myc mRNA were significantly increased in the colon cancer cells of *Adamts18* KO mice relative to WT littermates (Supplementary Table 2).

### *Adamts18* deficiency worsens colitis and alters cytokine expression profiles in DSS-induced colitis model

**Figure 5:**
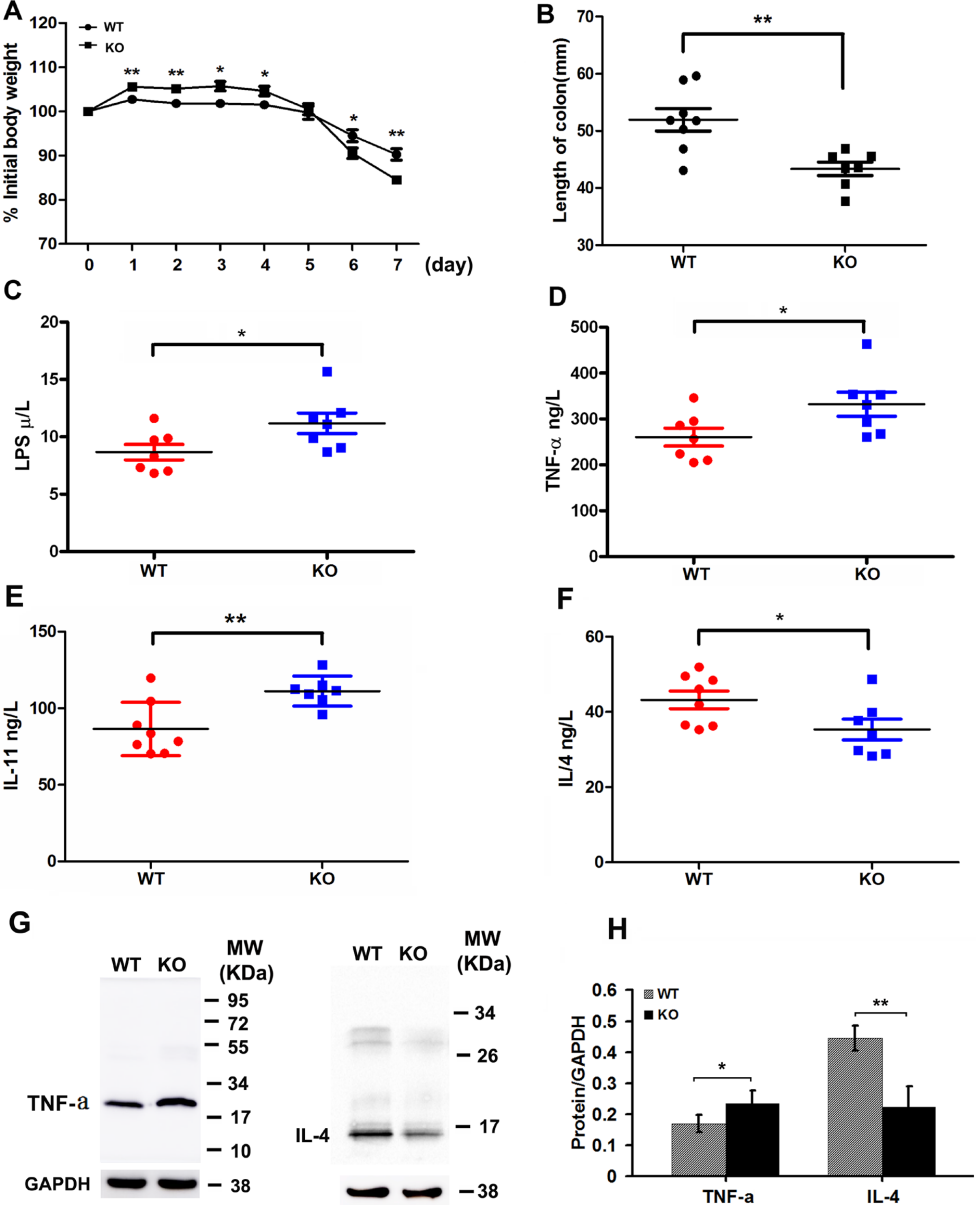
Adamts18 deficient mice are highly susceptible to DSS-induced colitis DSS colitis model was established in wild-type (WT) mice and *Adamts18* knock-out (KO) littermates by administration of 3% DSS solution in drinking water for 7 days. (**A**) The body weights of the WT and *Adamts18* KO mice were monitored during the study period. Weights are expressed as means of individual data relative to initial body weight of each animal. The *Adamts18* KO mice suffered from significant body weight loss from day 6. (**B**) Quantification of colon length of WT and *Adamts18* KO mice at time of sacrifice. (**C**–**F**) Sera lipopolysaccharides (LPS) and inflammatory cytokine assay. Concentrations of LPS (C), tumor necrosis factor-alpha (TNF-α) (D), interleukin (IL)-11 (E), and IL-4 (F) determined by sandwich ELISA. Each symbol represents one individual. Horizontal bars represent mean value. (**G**–**H**) Western blot analysis of the expression level of TNF-α and IL-4 in mice colitis tissues. Columns mean; bars, SD (*P < 0.05, **P < 0.01). These experiments are repeated independently at three times.

Inflammation has formed the key part of tumor microenvironment in the colon cancer. In DSS-induced colitis model, *Adamts18* KO mice showed severe bloody stools and suffered from significant body weight loss from day 6 compared to WT littermates (day 6: WT *vs*. KO, 94.5 ± 3.8% vs. 90.5 ± 3.2%, *P* = 0.048; day 7: WT *vs*. KO, 90.3 ± 3.6% vs. 84.5 ± 2.6%, *P* = 0.004) (Figure 5A). Additionally, significantly reduced colon length was also observed in *Adamts18* KO mice compared with WT littermates when mice were sacrificed at days 7 (WT *vs*. KO, 51.9 ± 5.6 *vs*. 43.4 ± 3.2 mm, *P* = 0.003) (Figure 5B). Pathological analysis demonstrated severer intestinal inflammatory infiltration, mucosa congestion and higher histological scores based on the degree of inflammation in *Adamts18* KO mice than in WT littermates (Supplementary Figure 2). Furthermore, mouse inflammatory cytokines PCR Array indicated that the mRNA expression of many downstream genes of activated mitogen activated protein kinase (MAPK) pathway, including *Tnf-α*, *Il17b, Il17f, Lta (TNF-b), Ccl1, Il11(Eotaxin)* and *Il1α*, were significantly up-regulated but the expression of anti-inflammatory cytokine IL-4 was down-regulated in colitis samples of *Adamts18* KO mice compared to those in WT mice (Supplementary Figure 3). The results of sandwich ELISA demonstrated that serum levels of LPS, TNF-α, and IL-11 were significantly elevated while IL-4 was decreased in *Adamts18* KO mice compared to those of WT mice [LPS (WT *vs*. KO, 8.7 ± 1.8 *vs*. 11.2 ± 2.4 U/L, *P* = 0.045); TNF-α (WT *vs*. KO, 260.4 ± 51.8 *vs*. 332.1 ± 69.4 ng/L, *P* = 0.049); IL-11 (WT *vs*. KO, 86.5 ± 17.4 *vs*. 111.2 ± 9.8 pg/mL, *P* = 0.006); IL-4 (WT *vs*. KO, 43.2 ± 6.6 *vs*. 35.3 ± 7.4 pg/mL, *P* = 0.048)] (Figure 5C–5F). Western Blotting analysis further confirmed that TNF-α was increased but IL-4 was decreased in colitis tissues of *Adamts18* KO mice when compared to those of WT control (Figure 5G and 5H).

### Increased p38MAPK and ERK activities in colon cancer cells of *Adamts18* deficient mice

**Figure 6:**
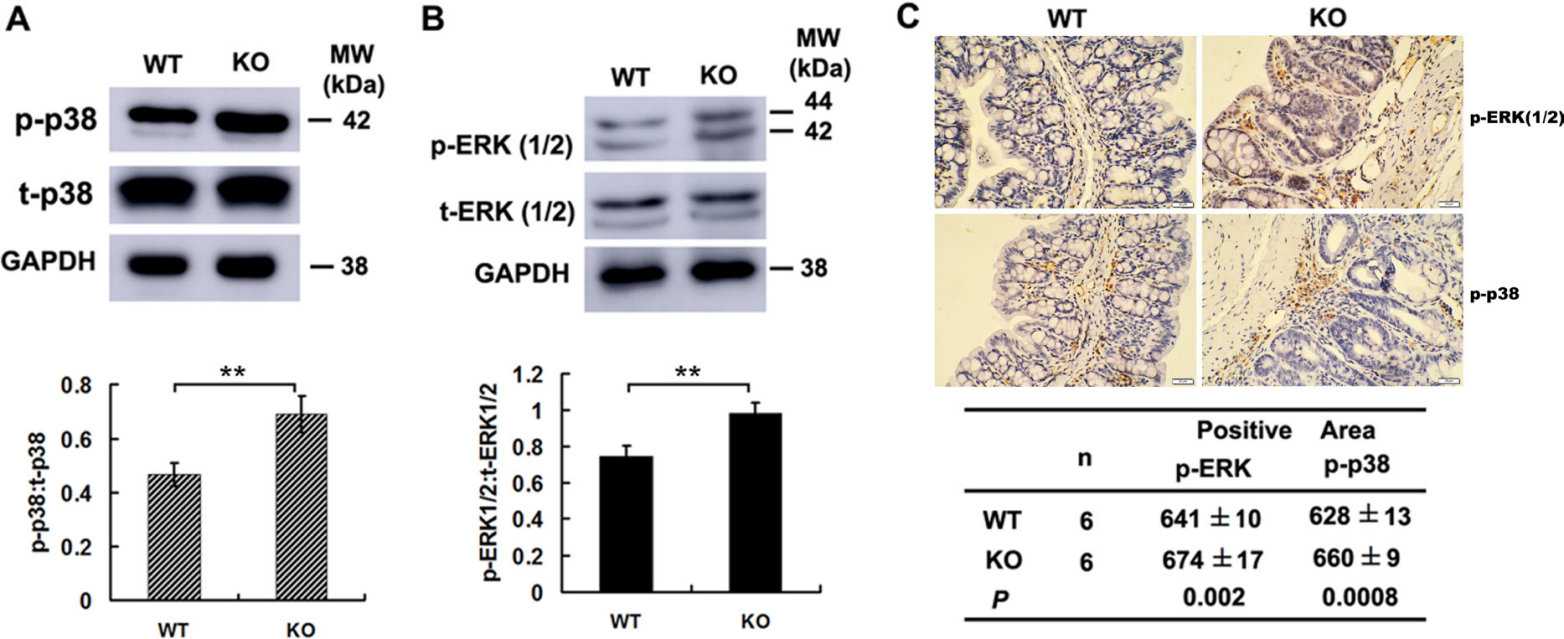
Increased p38MAPK and ERK1/2 activities in colon cancer cells of Adamts18 deficient mice (**A**–**B**) Western blot analysis of the activities of p38 MAPK (A) and ERK1 / 2 (B) in tumor tissues of mice. (**C**) Immunohistochemical analysis of the activities of ERK1/2 and p38MAPK in tumor tissues of mice. Brown, positive staining. The positive staining was calculated statistically using Image Pro Plus 6.0 software (Media Cybernetics Inc., Bethesda, MD, USA). Results are mean ± SD, **P < 0.01. Scale bar = 20 μm. These experiments are repeated independently at three times. WT, wide-type; KO, knock-out.

We further examined the activities of MAPK pathway in colon cancer cells. Western Blotting showed phosphorylated forms of both p38MAPK and ERK1/2 were significantly increased in tumor cells of *Adamts18* KO mice when compared with WT littermates (Figure 6A and 6B). Furthermore, the immunohistochemistry staining showed that the expression of phosphorylated p38MAPK and ERK1/2 were significantly increased in tumor tissues of *Adamts18* KO mice when compared with WT littermates [pERK positive area: WT *vs*. KO, 641 ± 10 *vs*. 674 ± 17, *P* = 0.002; p38MAPK positive area: WT *vs*. KO, 628 ± 13 *vs*. 660 ± 9, *P* = 0.0008) (Figure 6C).

### Decreased E-cadherin expression in intestinal epithelium of *Adamts18* deficient mice

**Figure 7:**
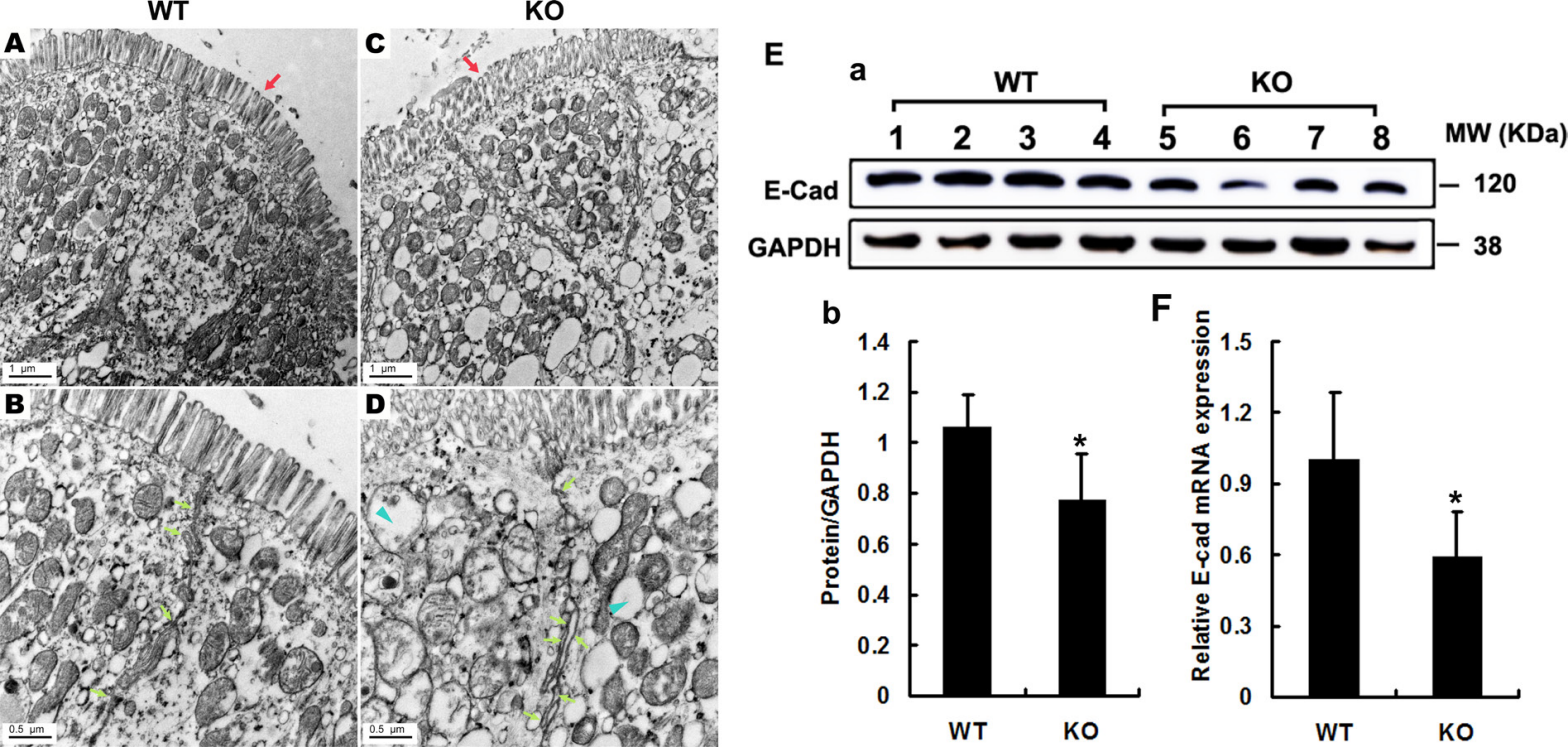
Electron microscopy analysis of the integrity of intestinal barrier (**A**–**D**) Intestinal villi in WT mice were intensive, in alignment (A) and tight junction were normal (B, arrows), whereas they were sparse, disorder (C, red arrows) and broadening intercellular space in *Adamts18* KO littermates (D, fluorescein arrow). In addition, cytoplasm in KO mice contains a large number of mitochondria vacuolar degeneration (D, blue arrowheads). (**E–F**) Expression levels of E-cadherin determined by Western blotting and RT-PCR and quantization of relative expression levels of the proteins and mRNA. Values are means of three replicated experiments. Statistical significance: *P < 0.05. WT, wide-type; KO, knock-out. Scale bar (A, C = 1 μm, B, D = 0.5 μm respectively).

Since the impairment of the intestinal barrier drives the pathogenesis of CAC [17], we then examined the colon ultrastructure by transmission electron microscopy (TEM). The results showed that intestinal villi were intensive and in alignment in WT mice (Figure 7A), whereas they were sparse, disrupted in *Adamts18* KO littermates (Figure 7C). Furthermore, broadened intercellular space between the intestinal epithelial cells (Figure 7C), and a large number of vacuolar degenerated mitochondria in the cytoplasm of intestinal epithelial cells (Figure 7D) were observed in *Adamts18* KO mice. We also examined the expressions of the pivotal molecules involved in the formation of intercellular tight junction including occludin, claudin-1 and E-cadherin in colon of mice. Western blotting showed that the expressions of occludin and claudin-1 in *Adamts18* KO mice were not significantly different from those in WT mice (Supplementary Figure 4). In contrast, *Adamts18* deficiency led to decreased E-cadherin expression in intestinal epithelium of KO mice (Figure 7E, a–b). We also checked the expression changes at mRNA levels by real-time RT-PCR and similar results were obtained (Figure 7F).

## DISCUSSION

Several studies have suggested the association between ADAMTS18 gene mutation (and inactivation) and colon cancer [11, 12]. However, the exact role of ADAMTS18 in colon cancer progression has not been reported. Here, we demonstrate for the first time that deficiency of this molecule increases the susceptibility of cells to mutagenic factor and facilitates inflammatory microenvironment. A combination of AOM and DSS is a well-established chemically induced CAC mouse model to understand the nuances of human colitis associated colon cancer. Combining a knockout model with this chemically induced CAC model is necessary to obtain efficient and reliable information regarding human colon carcinogenesis. However, AOM/DSS induced tumor formations are mainly located in the distal part of the colon. Thus, this experimental model only allows to seeing distal tumors, which are the limitations of the study.

In the AOM/DSS induced CAC model, aberrant activation of β-catenin signaling is critical for inducing intestinal tumorigenesis by activating its downstream target genes cyclinD1 and c-myc [16]. We observed that the expression of nuclear β-catenin together with cyclinD1 and c-myc were significantly enhanced in the colonic sections of *Adamts18* KO mice relative to WT littermates after AOM/DSS induction (Figure 4A–4F). Meanwhile, increased cell proliferation was observed in the tumors from *Adamts18* KO mice compared to WT littermates (Figure 3A). This suggested that *Adamts18* deficiency enhanced β-catenin-dependent intestinal tumorigenesis. This fact was further corroborated by reduced E-cadherin expression (~20%) in the colon tissue of *Adamts18* deficient mice (Figure 7E–7F). E-cadherin is the gene product of *CDH1* that is located with *ADAMTS18* on chromosome 16q and is critical for the integrity of cell-cell adhesion [18]. Downregulation of E-cadherin has been identified in numerous human cancers including colon cancer [18, 19]. In CAC, E-cadherin forms a complex with β-catenin and in combination with the actin cytoskeleton prevents nuclear translocation of β-catenin, thus regulating the activity of the Wnt/catenin signaling pathways [20]. In this regard, downregulation of E-cadherin in the colon tissue of *Adamts18* KO mice may promote nuclear translocation of β-catenin. Of note, interactions between metalloproteinases and E-cadherin have been highlighted in previous studies [21, 22]. For example, ADAM10 specifically cleaves the ectodomain of E-cadherin [21]. ADAM15 catalyzes and sheds soluble E-cadherin, which binding to the ErbB receptor in breast cancer cells [22]. Further, similar to our findings, the positive correlations between E-cadherin *(CDH1)* and *ADAMTS18* mRNA levels were found in human lymphoma samples [23], which may reflect the requirement of ADAMTS18 for E-cadherin processing in the cells.

Inflammation is a key component of the tumor microenvironment in colon cancer and the role of the ADMATS family members in inflammation has been reported in previous studies. ADAMTS12 has previously been implicated in intestinal inflammatory response by modulating normal neutrophil apoptosis [24]. In this study, we found that *Adamts18* deficiency promoted intestinal inflammation by increased activation of p38MAPK and ERK1/2 signaling pathway in mice. Indeed, several studies have showed that anti-tumor effects of ADAMTS8 [25] and ADAMTS12 [26] are closely associated with the suppression of ERK signaling. ERK /P38MAPK are required to regulate CRC cell proliferation and apoptosis [27] and their inhibition results in tumor growth reduction both *in vitro* and *in vivo* [28]. Thus, up-regulated ERK/p38MAPK signaling is one potential mechanism for increased colon tumorigenesis in *Adamts18* KO mice. In conclusion, our study demonstrates that *Adamts18* deficiency significantly promotes colon carcinogenesis and intestinal inflammation by enhancing β-catenin and ERK/p38MAPK signaling in the mouse model of AOM/DSS-induced CAC.

## MATERIALS AND METHODS

### Reagents

All reagents were obtained from Sigma-Aldrich (St Louis, MO) unless otherwise designated. Primary antibodies used in this study were listed in Supplementary Table 3.

### Generation of *Adamts18* deficient mice

To construct the targeting vector, a plasmid containing two Loxp sites, a Frt (flippase recognition target site) flanked PGK/Neo cassette, and a herpes simplex virus thymidine kinase (HSV-TK) cassette was used as the cloning vector. The homologous DNA fragments were excised from BAC clone bMQ-54M16 (129S7/AB2.2, Sanger Center) containing *Adamts18* gene and inserted into the plasmid. After the targeting vector was constructed, a Frt flanked PGK/Neo cassette and exons 5–6 of murine *Adamts18* gene were flanked by two LoxP sites, and the HSV-TK cassette was put in the flanking region of the 5′ arm. The targeting vector was linearized and electroporated into murine embryonic stem (ES) cells derived from strain 129Sv, and neomycin-resistant clones were screened for correct homologous recombination by Southern blot with NcoI-digested genomic DNA isolated from ES cell clones. The homologous recombination was confirmed by another Southern blotting with MfeI-digested genomic DNA from two ES cell clones. After karyotype analysis, one of the ES cell clones harboring the homologous recombination was microinjected into C57BL/6J blastocysts, and chimeras that transmitted the LoxP flanked (exons 5–6) *Adamts18* gene allele through the germ line were obtained. Heterozygotes with deletion of exons 5–6 of *Adamts18* gene were obtained by crossing the *Adamts18* floxed mice with a transgenic mouse carrying a Sox2-Cre (on a C57BL/6 background) transgene, which is expressed in all tissues including oocytes in mouse. Homozygotes with deletion of exons 5–6 of *Adamts18* gene were obtained by mating between heterozygotes. Genomic DNA extracted from mouse tails was used as template for PCR identifying genotypes. The sequences of primers used for genotyping, the combination of the primers and the expected PCR product size for *Adamts18* floxed and *Adamts18* KO mice are list in Supplementary Table 4. Expression of *Adamts18* mRNA and protein was determined by RT-PCR and Western blot, respectively. Primers specific to *Adamts18* exon 4 (E_4_F) and exon 6 (E_6_R) were used for RT-PCR (Supplementary Table 4). Mice were maintained in a specific pathogen-free facility at East China Normal University (ECNU). All procedures in animal experiments were approved by the Institutional Animal Care and Use Committee of ECNU.

### Animal models

#### *AOM/DSS*-induced CAC in mice

Male mice aged 8 weeks were injected intraperitoneally with 10 mg/kg colonic carcinogen AOM (Cat. No. A5486; Sigma Chemical, St. Louis, MO, USA). Then, 2.5% DSS with a molecular weight of 36000–50000 (Cat No.160110; MP Biomedicals LLC, Aurora, OH, USA) was given in the drinking water over seven days, followed by two weeks of regular water. This cycle was repeated three times. Fourteen days after the last round of DSS, animals were sacrificed by cervical dislocation under narcosis and colon tumors were photographed and then fixed overnight in 10% formalin and paraffin-embedded for histology analysis. The pathological degree of induced colitis and dysplasia were scored by a specialist in a double-blind fashion according to the criteria reported previously [17].

### DSS-induced colitis in mice

Male mice aged 8 week were divided into two groups, receiving 2.5% (w/v) DSS in water for 7 days. The mice were checked each day for the development of colitis by monitoring their body weight and diarrhea. All mice were sacrificed by cervical dislocation under narcosis after the experiment and the colons were taken for histological analysis.

### Histological, immunohistochemical (IHC), and *In situ* TUNEL analysis

Colon specimens were fixed immediately in 10% buffered formalin and subsequently embedded in paraffin. Standard hematoxylin-eosin (HE) sections were used for pathological analysis. For IHC analysis, slides were incubated with indicated primary antibodies in PBS containing 1% BSA and 10% goat serum overnight at 4°C. Then, streptavidin-HRP was added, and the sections were finally stained with DAB (3, 3 -diaminobenzidine) HRP substrate. The *in situ* TUNEL analysis was performed using TUNEL apoptosis detection kit (Millipore, USA) according to the manufacturer's instructions. The number of Ki-67-positive tumor cells and the total number of tumor cells were enumerated in six microscopic fields of a randomly selected tumor and the mean value was calculated as the percentage of Ki-67- positive tumor cells (Ki67 labeling index). For analysis of apoptosis, the percentage of apoptotic cells (apoptotic index) was determined by calculating the positive cell number: total cell number × 100.

### Western blotting and Sandwich ELISA

For Western blot analysis, equal amount of protein homogenates were separated on a 12% SDS–PAGE under reducing conditions and then transferred onto a polyvinylidene difluoride (PVDF) membrane. The membrane was blocked in blocking buffer [PBS, 0.5% Tween-20, and 5% non-fat dry milk powder or 1% bovine serum albumin (BSA)], and then incubated with primary antibody for 1 hour at room temperature (RT). After washing, the membrane was incubated with horseradish peroxidase (HRP)-conjugated secondary antibody for 1 hour at RT. The immunoreactive bands were visualized with enhanced chemiluminescence (ECL) kit according to the manufacturer's instructions. Serum LPS and cytokine TNF-α, IL-11, IL-4 were measured with ELISA kits from R&D system according to the manufacturer's instructions.

### Electron microscopy

Mouse colon samples were fixed in 2.5% glutaraldehyde in 0.1 M sodium cacodylate buffer, then dehydrated, infiltrated with EPON-812 resin and embedded in capsules. The enclosed tissues were cut on a Reichert Ultracut 5 ultramicrotome in super thin sections. The sections was collected onto formvar-coated slot grid, and analyzed using a Zeiss EM-10 transmission electron microscope with digital acquisition.

### Statistical analysis

All numerical data are presented as mean ± standard deviation (SD). Each experiment was repeated at least three times. The statistical significance of differences between groups was evaluated by the unpaired two-tailed Student's *t* test and indicated with ***P < 001, **P < 0.01, *P < 0.05.

## SUPPLEMENTARY MATERIAL TABLES AND FIGURES


