# Supplementary material for: Adamts18 deficiency promotes colon carcinogenesis by enhancing β-catenin and p38MAPK/ERK1/2 signaling in the mouse model of AOM/DSS-induced colitis-associated colorectal cancer
Source: Oncotarget. 2017 Jan 27;8(12):18979–90. doi: 10.18632/oncotarget.14866 (PMC5386663; doi:10.18632/oncotarget.14866)
Supplement: Supplementary file 1 [file oncotarget-08-18979-s001.pdf]

Adamts18 deficiency promotes colon carcinogenesis by enhancing β-catenin and p38MAPK/ERK1/2 signaling in the mouse model of AOM/DSS-induced colitis-associated colorectal cancer

Supplementary Materials

Methods

Inflammatory genes RT² PCR array

Expression of inflammatory genes was evaluated with the mouse RT²*Profiler* PCR Inflammatory Cytokines and Receptors Array (SABiosciences). Two micrograms of RNA were used for cDNA synthesis with the RT² First Strand Kit (SABiosciences). The RT²*Profiler* array was probed according to the manufacturer's protocol using the Profiler PCR Array System and SYBR Green/Fluorescein qPCR Master Mix (SABiosciences) in an ABI 7900 sequence analyzer (Applied Biosystems). Gene expression was compared with the dedicated Web-based software package (http://www.superarray.com/pcr/arrayanalysis.php), which automatically performs all ΔΔCt based fold-change calculations from the specific uploaded raw threshold cycle data.

Angiogenesis analysis

Tumor tissues were fixed in 10% formaldehyde in phosphate-buffered saline (PBS), pH 7.4, dehydrated and embedded in paraffin and sectioned with a microtome. Intratumoral vascular density was assessed by staining with goat anti-mouse CD31 antibody (Santa Cruz Biotechnology Inc., CA). Microvessel positive area (MPA) was determined by light microscopy in areas of invasive tumor containing the highest numbers of capillaries and microvessels per area. MPA was expressed as the positive staining of microvessel per high-power field.

Real-time reverse transcription-polymerase chain reaction (RT-PCR)

Total RNA was extracted from colon tissues using TRIzol (Invitrogen Corp.). The RNA was treated with DNase according to the manufacture's instructions (Invitrogen Corp.). A total of 1 µg of RNA was reverse-transcribed with SuperScript™ III First-Strand cDNA Synthesis Kit (Invitrogen Corp.). The mRNA levels were determined using real-time RT-PCR sequence detection (7500 Real Time System; Applied Biosystems). Primers are listed in Supplementary Tables 1 and 2. Levels of mRNA expression were normalized to actin mRNA.

Supplementary Table 1: The expressions of angiogenesis-related ADAMTSs determined by RT-PCR

Murine	Primers	bp	Wild-type [RQ($2^{-\text{Delta Delta Ct}}$)] ($n$ = 7)	Knockout [RQ($2^{-\text{Delta Delta Ct}}$)] ($n$ = 7)	P
ADAMTS1	Forward: 5′-GGCATTGGCTACTTTTTCGTCT-3′ Reverse: 5′-TCTTCTTGCATGTGGAACCGT-3′	184	0.99 ± 0.09	0.98 ± 0.14	0.957
ADAMTS8	Forward: 5′-AACCGTGATGTGATAATCCAATG-3′ Reverse: 5′-AGTCAGTGTGGTTGTAGGCATTA-3′	161	1.13 ± 0.09	1.05 ± 0.29	0.581
ADAMTS12	Forward: 5′-ACAAGCATCTTATGAAGGACCACA-3′ Reverse: 5′-GTGGCACTTCTTAGCAGGGTCT-3′	170	0.97 ± 0.13	1.09 ± 0.25	0.297
β-Actin	Forward: 5′-CCACCATGTACCCAGGCATT-3′ Reverse: 5′-AGGGTGTAAAACGCAGCTCA-3′	253			

Data are expressed as mean ± SEM. Bp, base pair.

Supplementary Table 2: The expressions of β-catenin-related signaling molecules and E-cadherin determined by RT-PCR

Murine	Primers	bp	Wild-type [RQ($2^{-\text{Delta Delta Ct}}$)] ($n$ = 7)	Knockout [RQ($2^{-\text{Delta Delta Ct}}$)] ($n$ = 7)	P
C-myc	Forward: 5′-TCGCCCAAATCCTGTACCTC-3′ Reverse: 5′-TTTCTTCCTCATCTTCTTGCTCTT-3′	172	0.79 ± 0.32	1.38 ± 0.31	0.021
Cyclin D1	Forward: 5′-GCTACCGCACAACGCACTT-3′ Reverse: 5′-CGCAGGCTTGACTCCAGAA-3′	103	0.32 ± 0.11	0.86 ± 0.25	0.012
E-cadherin	Forward: 5′- GTCCTGCCAATCCTGATGAAAT-3′ Reverse: 5′- CAGAACCACTGCCCTCGTAAT-3′	123	0.52 ± 0.15	0.31 ± 0.10	0.031
β-Actin	Forward: 5′-CCACCATGTACCCAGGCATT-3′ Reverse: 5′-AGGGTGTAAAACGCAGCTCA-3′	253			

Data are expressed as mean ± SEM. Bp, base pair.

Supplementary Table 3: Antibodies used in this study

NO.	Product Name	Application	Source
1	Anti-Ki67 antibody	IHC-P	Abcam (ab15580)
2	Anti-beta Catenin antibody [E247]	IHC-P	Abcam (ab32572)
3	Anti-c-Myc antibody [Y69]	IHC-P	Abcam (ab32072)
4	Anti-Cyclin D1 antibody [EPR2241]	IHC-P	Abcam (ab134175)
5	Anti-TNF alpha antibody	WB	Abcam (ab9635)
6	Anti-IL4 antibody [BVD4-1D11]	WB	Abcam (ab11524)
7	p38 MAPK Antibody	WB	CST (#9212)
8	Phospho-p38 MAPK (Thr180/Tyr182) Antibody	WB, IHC-P	CST (#9211)
9	p44/42 MAPK (Erk1/2) Antibody	WB	CST (#9102)
10	Phospho-p44/42 MAPK (Erk1/2) (Thr202/Tyr204) Antibody	WB, IHC-P	CST (#9101)
11	Anti-E Cadherin Antibody	WB	Abcam (ab15148)
12	Anti-Occludin Antibody	WB	Abcam (ab154221)
13	Anti-Clandin1 Antibody	WB	Abcam (ab79404)
14	Anti-ADAMTS18 antibody- C-terminal	WB, IHC-P	Abcam (ab135728)

Supplementary Table 4: Primers used in the generation of *Adamts18* deficient mice

Primer	Sequence
p1	5′- TCTGCTGGAAGAAGAACCTGTAAGTGGAA-3′
p2	5′- TTTCCTCCTTCCTTTGCTGCCTTGTCTG-3′
p3	5′- GGTGTTGGTTGGTATAGCTGAGCTGGAAA-3′
E_4F	5′- TCCTCATCTCACCGCTACCTCA-3′
E_6R	5′- GGTCCCATCTTTGAACAGGCTA-3′

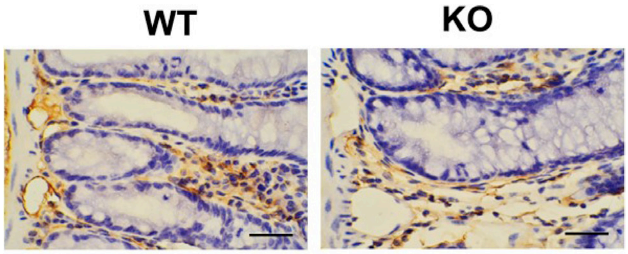

	n	Positive Area (CD 31)
WT	4	704 ± 55
KO	4	695 ± 46
P		0.805

Supplementary Figure 1: Microvessel CD31 positive staining in each group (*n* = 4/group). Microvessel positive area (MPA) was determined by light microscopy in areas of invasive tumor containing the highest numbers of capillaries and microvessels per area. MPA was expressed as the positive staining of microvessel per high-power field. Data are expressed as mean ± SEM. WT, wild-type mouse; KO, knockout mouse. Scale bar = 50 µm

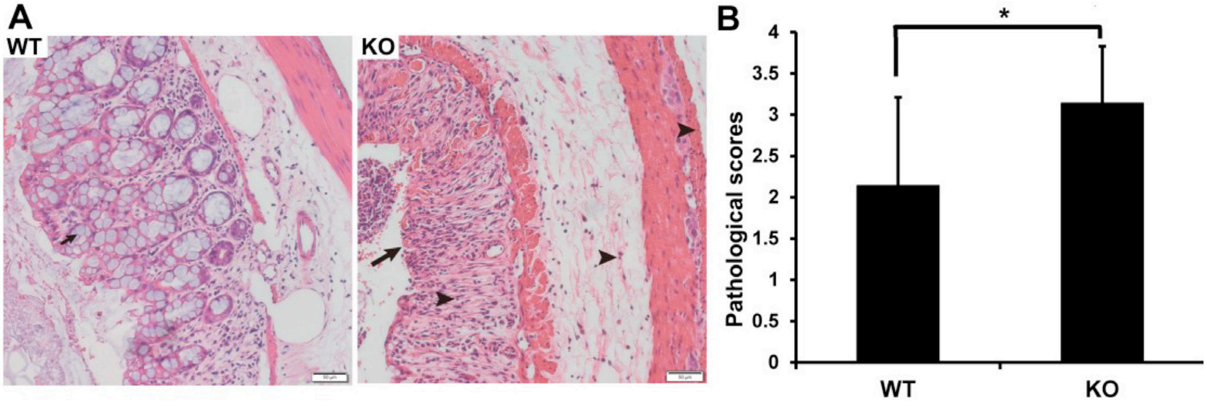

Supplementary Figure 2: Histopathological changes (Left) and histological score (right) of colitis in WT and *Adamts18* KO mice. Big arrowheads (KO), mucosal atrophy; Triangular arrowheads (KO), inflammatory cell infiltration; Small arrowheads (WT), goblet cells. Histology score was performed as described in methods. Data are represented as mean and standard deviation of three independent experiments (**P* < 0.05). Scale bar = 50 µm

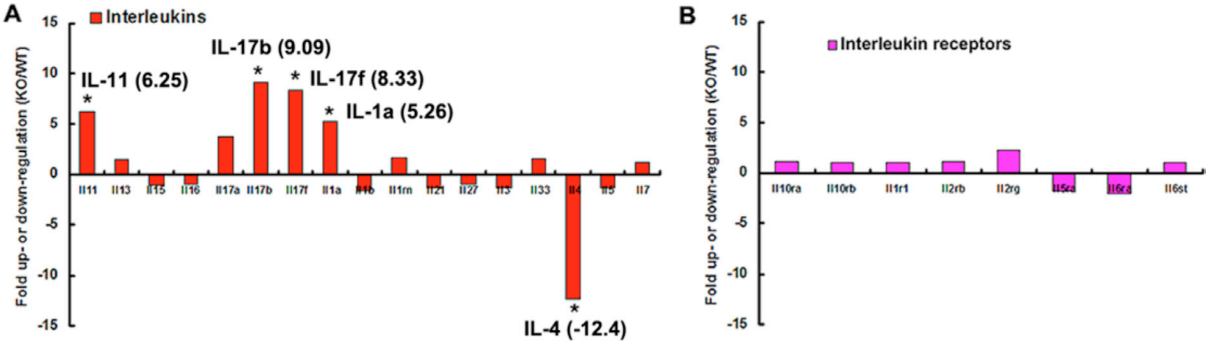

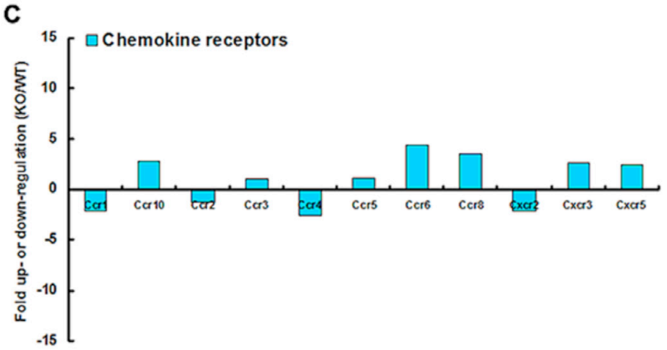

F

Name	Fold	Function
TNF	11.07	MAPK
IL-17b	9.09	MAPK
IL-17f	8.33	MAPK
Lta	7.53	NF-KappaB
Ccl1	7.38	Chemokine
IL-11	6.25	ERK
IL-1a	5.26	MAPK
Csf3	-5.86	Cytokine
IL-4	-12.4	Anti-inflammatory

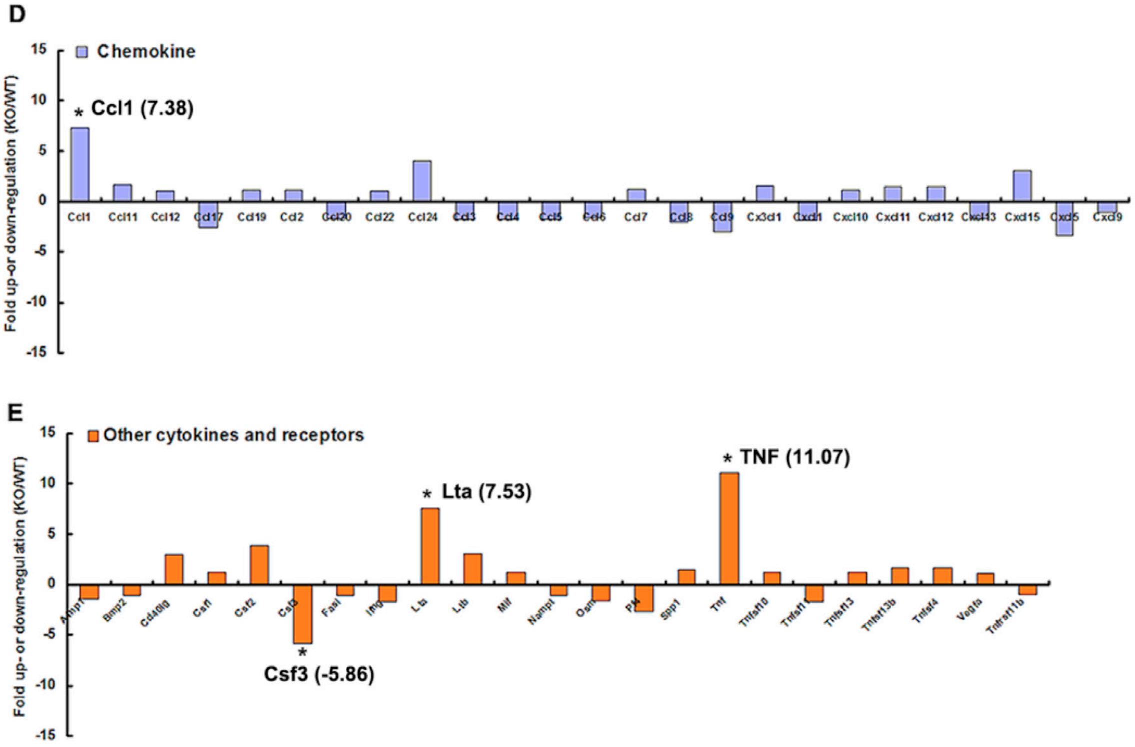

Supplementary Figure 3: Fold changes of mouse inflammatory cytokines and receptors determined by PCR Array as described in methods (*n* = 7/group). (**A**) Interleukins. (**B**) Interleukin receptors. (**C**) Chemokine receptors. (**D**) Chemokine. (**E**) Other chemokines and receptors. (**F**) Summary of inflammatory cytokines. Fold-Change (2^(- Delta Delta Ct)) is the normalized gene expression (2^(- Delta Ct)) in the Test Sample divided the normalized gene expression (2^(- Delta Ct)) in the Control Sample.

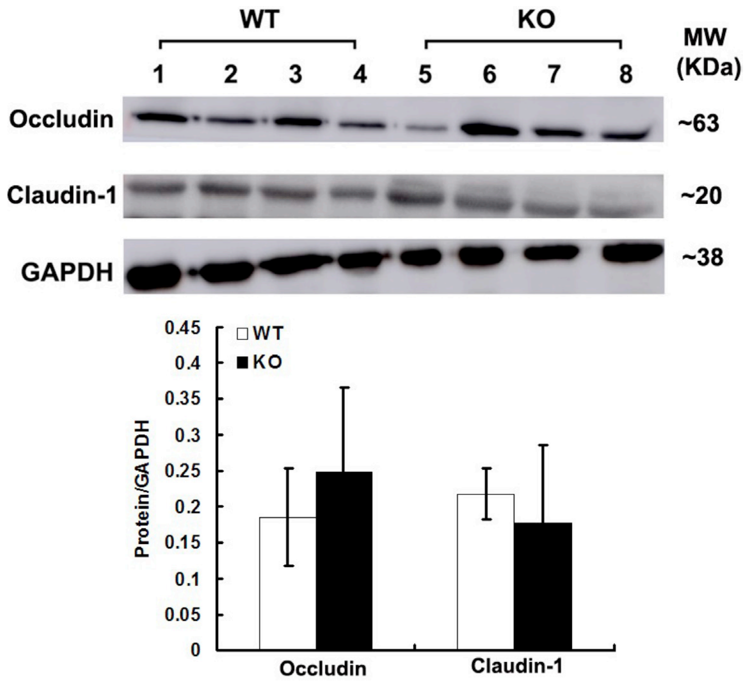

Supplementary Figure 4: Expression levels of occluding and claudin-1 in colon epithelium of mice determined by Western blotting. The expression level of GAPDH was used as the loading control. WT, wide-type mice; KO, knockout mice. Values are means of three replicated experiments.
